# The Impact of the COVID-19 Pandemic on the Lives of People With Gender Dysphoria

**DOI:** 10.3389/fpubh.2022.878348

**Published:** 2022-07-06

**Authors:** Fernanda Guadagnin, Dhiordan Cardoso da Silva, Karine Schwarz, Anna Paula Villas Bôas, Maria Inês Rodrigues Lobato

**Affiliations:** ^1^Clinical Hospital of Porto Alegre, Porto Alegre, Brazil; ^2^Department of Psychiatry, Gender Identity Program at Hospital de Clínicas de Porto Alegre, Porto Alegre, Rio Grande do Sul, Brazil; ^3^Postgraduate Program in Medical Sciences: Endocrinology, Federal University of Rio Grande do Sul (UFRGS), Porto Alegre, Brazil; ^4^Postgraduate Program in Psychiatry and Behavioral Sciences at the Federal University of Rio Grande do Sul, Porto Alegre, Brazil

**Keywords:** Gender Dysphoria, COVID-19, health monitoring, vulnerability, transgender

## Abstract

**Objective:**

To analyze the impact on the psychological and social aspects caused by the COVID-19 pandemic in individuals diagnosed with Gender Dysphoria (GD).

**Methods:**

Google Forms inventory was sent via WhatsApp, including qualitative and quantitative questions evaluating three life dimensions denominated as Sociodemographic, Economic, and COVID-19 pandemic. It was applied in two periods: At the beginning of the pandemic (June-2020) (P1) and one year later (June-2021) (P2). The inventory also included questions about economic dimensions before the pandemic for individual comparison purposes (P0). 48 individuals (28 transsexual women, 20 transsexual men) participated in both periods.

**Results:**

77.1% (*n* = 37) lived in Rio Grande do Sul, 50.0% (*n* = 24) refereed incomplete high school; Monthly Income increased significantly between the periods (P0) and (P1). Emergence aid approval was significantly higher in (P2), 56.3% (*n* = 27), compared to (P1), 39.6% (*n* = 19). A statistically significant difference was detected in the feeling of depression in the (P2) among the cases that requested Emergency Aid.

**Conclusion:**

The studied population presented deterioration regarding their condition of social vulnerability in relation to formal employment, access to health services, and mental health.

## Introduction

Gender Dysphoria (GD-DSM-5) is defined by the incongruence between a person's gender and their sexual body appearance and is characterized by intense psychological suffering. Since 1998 (1) –after regulation of the Federal Council of Medicine in 1997 (Resolution. 1. 482)–the Hospital de Clínicas de Porto Alegre (HCPA) has been providing hospital care through a multidisciplinary team for people with GD and, since then, more than 1,000 individuals have been attended to and PROTIG has already performed about 354 Gender Affirmation Surgeries (GAS).

In December 2019, a new coronavirus named as SARS-CoV-2 was identified in Wuhan, China. From then until now, the so-called COVID-19 pandemic has already caused the death over a million people worldwide. Handling the COVID-19 pandemic has changed virtually all human activities. Hospitals and health care systems have had to expand and prioritize medical care for critically ill patients and had to suspend or reduce all non-emergency or non-time-sensitive medical care, focusing on the control of clinical conditions secondary to COVID-19 infection.

Following these events, in March 2020, HCPA suspended/reduced all non-critical outpatient care and all elective surgeries due to the COVID-19 pandemic. At the same time Brazil began restricted measures of social distancing, and as a consequence, the suspension of commercial, educational, and social activities were adopted.

From March to August 2020, PROTIG suspended medical assistance, but continued to send prescriptions by email when requested. As of August 2020, the multidisciplinary team started to provide individual and group follow-up by telephone and video call.

The assistance currently provided at PROTIG consists of a diagnostic evaluation by a screening psychiatrist, after which, the patient will be referred for consultations with a social assistant, psychologist, and endocrinologist. After this initial period, the patient is invited to participate in monthly group therapy and will have individual consultations with psychiatry, endocrinology and social assistance services every 3 months or whenever necessary until GAS is performed.

In April, 2020, the Brazilian government granted an Emergency Aid, a benefit established by Law No. 13,982/2020, which provided the transfer of approximately R$ 600.00/monthly (around $107-US dollars/month) to informal and low-income workers, individual microentrepreneurs, and also individual tax payers of the National Institute of Social Security. The aid was created to assist in social protection measures and mitigate the economic crisis resulting from the effects caused by COVID-19 in Brazil. This resource was paid in five installments of R$ 600.00 or 1,200.00 for single-parent, and then extended until December 31, 2020, in up to four installments of R$ 300.00 or 600.00 each. The Emergency Aid was extended again until October 2021 at lower amounts and has been widely discussed between government authorities and the population about the need to maintain it, considering the economic scenario in Brazil.

GD population still has different social vulnerabilities despite they have been gaining some recognition of their political, social, gender, and sexual affirmation rights in recent years (2). GD population's social vulnerabilities may begin inside their families with some situations causing them to leave home and school, leading to poor professional qualifications. Apart from their families, they also face unfavorable social environment which can have consequences on their mental health. This adverse environment experienced by transgnder people corresponds to the description of Minority Stress (3). This model of stress was coined in 1995 by Ian Meyer, and is defined as psychological stress experienced by minority groups suffering prejudice and its consequences. This article aims to evaluate the clinical, social, and economic consequences on the GD population caused by the COVID-19 pandemic during the suspension of PROTIG assistance.

## Methodology

The 143 patients who met the diagnostic criteria for GD (DSM-5), evaluated by a team of PROTIG psychiatrists and were in active care during the year 2020/2021 received a link, by WhatsApp, with 24-questions questionnaire created on the online platform Google Forms, that included the Sociodemographic dimension (gender identity, sexual orientation, place of residence, race, and schooling), the Economic dimension (formal or informal work and income), the COVID-19 pandemic dimension (symptoms of COVID, confirmation of positive or negative for infection, access to health services, difficulties in accessing health services due to GD status, family relations before and after the pandemic, family members with COVID-19 and/or death of relatives), and mental health symptomatology (anxiety, depression, and irritability) due to the pandemic. This questionnaire was elaborated by the PROTIG's multidisciplinary team after a consensus about the life dimensions that need to be included based on our knowledge of the GD population. The questionnaire was sent in two periods: in June 2020 (P1) and 1 year later, June 2021 (P2). The questionnaire also included economic data from the pre-pandemic period, referred to as (P0). For comparative purposes, three periods were considered in the analysis of the economic dimension. The questionnaire also included some open-ended questions.

Out of the 143 patients studied, 77 responded at (P1), 63 responded at (P2), and 48 patients responded in both time periods, which was the final sample. There were no exclusion criteria. The drop in the number of participants is probably due to difficulty in accessing the internet or lack of interest in the research.

This study was approved by the Research Ethics Committee of the HCPA, (protocol no. 2019-0115). All participants were informed about the objectives of this research and then invited to participate after signing a written consent form.

The statistical analysis of data was performed with the Statistical Package for Social Sciences version 25.0 (SPSS Inc., Chicago, IL, USA, 2018) for Windows, and initially the data were organized in a Microsoft Excel^®^ spreadsheet. The results were presented by descriptive statistics with absolute and relative distributions (*n*-%). The analysis involving the comparison of categorical variables between initial and final evaluations, including the periods before and during the pandemic, were performed by the McNemar test. Pearson's chi-square test was used to compare categorical variables between independent groups.

## Results

### Sociodemographic Data Dimension

The sample consisted of 48 GD individuals, 58.3% (*n* = 28) transgender women (TW) and 41.7% (*n* = 20) transgender men (TM). Regarding sexual orientation, 54.2% (*n* = 26) feel attracted exclusively to men and 29.2% (*n* = 14) exclusively to women. Most participants were white, 70.8% (*n* = 34), and resided in the state of Rio Grande do Sul, 77.1% (*n* = 37). Regarding schooling level, 24 individuals had incomplete high school (50.0%), and 18 had incomplete higher education (37.5%) (Table 1).

**Table 1:** Sociodemographic profile at baseline.

	**Participants *n* (%)**
**Gender**
Transgender women	28 (58.3%)
Transgender men	20 (41.7%)
**Education**
Incomplete elementary School	08 (16.7%)
Incomplete high school	22 (45.8%)
Incomplete undergraduate	18 (37.5%)
**Sexual orientation**
Attraction to men	26 (54.2%)
Attraction to women	14 (29.2%)
Attraction to men and women	4 (8.3%)
Others	4 (8.3%)
**State where they live**
RS	37 (77.1%)
SC	4 (8.3%)
DF	3 (4.2%)
SP	2 (4.2%)
PR	2 (4.2%)

### Economic Dimension

Regarding economic data, formal, informal employment, and unemployment did not show statistically significant differences between the three periods. It was observed that the cases with formal employment in (P0) were 41.7% (*n* = 20), reducing to 39.6% (*n* = 19) in (P1), and reaching 37.5% (*n* = 18) in (P2). The number of unemployed, compared to (P0) 25.0% (*n* = 12), increased in (P1), 35.4% (*n* = 17) followed by a reduction in (P2), 29.2% (*n* = 14). Note that, the population with GD had a high degree of informal work or previous unemployment (58.3%), unrelated to the pandemic (Table 2).

**Table 2:** Absolute and relative distribution for work and monthly income in the segments before (P0), initial (P1), and final (P2).

**Work**	**Ratings/ segment (*****n*** **=** **48)** ^**A**^					
	**Before P0**		**Initial P1**		**Final P2**	
	***n***	**%**			***N***	**%**
**Work**
Formal	20	41.7	19	39.6	18	37.5
informal	16	33.3	12	25.0	16	33.3
Unemployed	12	25.0	17	35.4	14	29.2
P (P0-P1)	0.082					
P (P0-P2)	0.522					
P (P1-P2)	0.388					
**Monthly income**
No income	10	20.8	17	35.4	14	29.2
From R$1,045.00 to 3,135.01	38	79.2	31	64.6	33	68.8
R$ 3,135.01 to 5,225.00	-	-	-	-	-	-
* *p^B^ (P0-P1)	0.016					
p^B^ (P0-P2)	0.158					
p^B^ (P1-P2)	0.429					

^A^, Results obtained based on the total sample; ^B^, McNemar Browker test; *, significant for p < 0.05.

The social vulnerability experienced by the population with GD was reinforced in the findings regarding their monthly income. The results show a significant difference between the periods (P0) and (P1) [McNemar B = 8,412; *p* = 0.016], indicating a representative increase in the number of patients with an increase in their monthly income: (P0), 20.8% (*n* = 10) vs. (P1), 35.4% (*n* = 17). These paradoxical findings are related to the obviously precarious economic situation that GD people have in their everyday lives and the positive difference that the Emergency Aid provided during the pandemic. Moreover, no statistical difference was found between (P0), (P1), and (P2): [McNemar B = 8. 412; *p* = 0.016], [McNemar B = 3,333; *p* = 0.189] and [McNemar B = 1,692; *p* = 0.429] in their monthly income, even with the decrease in formal and informal employment (Table 3).

**Table 3:** Absolute and relative distribution for the request and approval of emergency assistance and for treatment and surgical procedure in the initial (P1) and final (P2) segments.

**Emergency aid**	**Ratings/ segment (*****n*** **=** **48)** **A**				
	**Initial P1**		**Final P2**		**B ^**P**^**
	***N***	**%**	***n***	**%**	
**Request**
Yes	26	54.2	28	58.3	
No	22	45.8	20	41.7	0.867
**Approval**
Yes	19	39.6	27	56.3	
No	29	60.4	20	41.7	*0.009
I haven't received a response yet			1	2.1	
**Hormone treatment**
Feminizing hormone	23	47.9	21	43.8	0.902
Masculinizing hormone	20	41.7	20	41.7	
none	5	10.4	7	14.6	

^A^, Results obtained based on the total sample; ^B^, McNemar Browker test; *, significant for p < 0.05.

The request for Emergency Aid was similar between (P1), 54.2% (*n* = 26) and (P2), 58.3% (*n* = 28) [McNemar B = 1,068; *p* = 0.867]. However, there was evidence that the rate of aid approval was significantly higher in (P2), 56.3% (*n* = 27), when compared to (P1), 39.6% (*n* = 19) [McNemar B =8.422; *p* = 0.009] (Table 3).

## Covid-19 Pandemic Dimension

### Emergency Aid and Feelings

According to the results, a statistically significant difference was detected in the feeling of depression in (P2) [X 2 1 = 6,696; *p* = 0.005], so that, the perception of feeling depressed was significantly higher among the cases that requested Emergency Aid, 50.0% (*n* = 14), when compared to those that did not request it, 10.0% (*n* = 2) (Table 4).

**Table 4:** Absolute and relative distribution for “emergency assistance” in the initial (P1) and final (P2) segments according to anxious, depressed, and angry feelings.

**Feelings**	**Emergency aid P1** ^**A**^				**Emergency aid P2** ^**B**^				***p-*value**
	**Yes (*****n*** **=** **26)**		**No (*****n*** **=** **22)**		**Yes (*****n*** **=** **28)**		**No (*****n*** **=** **20)**		
	***n***	**%**	***n***	**%**	***n***	**%**	***n***	**%**	
									0,874
**Anxiety**	15	57.7%	10	45.5%	19	67.9%	10	50.0%	
No	11	42.3%	12	54.5%	9	32.1%	10	50.0%	
X ${}_{\text{calc};}^{2}$*p*	0.309; *p* = 0.578				0.889; *p* = 0.343			
**Depression**									*0.009
Strong	8	30.8%	7	31.8%	14	50.0%	2	10.0%	
No	18	69.2%	15	68.2%	14	50.0%	18	90.0%	
X ${}_{\text{calc};}^{2}$*p*	0.006; *p* > 0.999				6.696; *p = 0.005			
**Anger**									0.588
Strong	14	53.8%	9	40.9%	13	46.4%	8	40.0%	
No	12	46.2%	13	59.1%	15	53.6%	12	60.0%	
X ${}_{\text{calc};}^{2}$*p*	0.365; *p* = 0.576				0.022; *p* = 0.883			

^a^, Results obtained based on the total of each emergency aid response category; ^b^, X ${}_{\text{calc}}^{2}$ -Pearson chi-square test of association; *, significant for p < 0.05.

The significant difference was also confirmed in the comparison between P1 and P2; [X 2 1 = 7,315; *p* = 0.009] where the proportion of cases with feeling depressed was higher in the final period, 50.0% (*n* = 14), compared to the initial period 30.8% (Table 4).

There were no statistical differences between P1 and P2 concerning anger and anxiety (Table 4).

### Family Relations and Access to Health Services

The perception of good family relationships prevailed in all periods (P0), 97.9% (*n* = 47) and (P1), 87.5% (*n* = 42), and (P2) 95.8% (*n* = 46). However, we found a small group in the study with bad family relationships that did not seek health services to the same degree compared to the group with good family relationships: (P1), 11.4% (*n* = 5); and P2, 2.7% (*n* = 1) [X 2 1 = 3,566; p=0.038]. We also found a high number of GD people facing difficulties in their search for health services regarding GD prejudice / discrimination. P1: 33.3% (*n* = 16) and P2: 37.5% (*N* = 18) (Table 5).

**Table 5:** Absolute and relative distribution for “access to health services” in the initial (P1) and final (P2) segments according to the family relationship.

**Family relationship**	**Access to the health service- sought health service-symptoms covid** ^**A**^												***B* ^P^**
	**Segment before P0**				**Initial segment P1**				**Final P2 segment**				
	**Yes (*****n*** **=** **4)**		**No (*****n*** **=** **44)**		**Yes (*****n*** **=** **4)**		**No (*****n*** **=** **44)**		**Yes (*****n*** **=** **11)**		**No (*****n*** **=** **37)**		*0.045
	**N**	**%**	**n**	**%**	**n**	**%**	**n**	**%**	**n**	**%**	**n**	**%**	
Good	4	100.0	43	97.7	3	75.0	39	88.6	10	90.9	36	97.3	*0.038
Bad			1	2.3	1	25.0	5	11.4	1	9.1	1	2.7	
X ${}_{\text{calc};}^{2}$*p ^*C*^*	0.284; *p* = 0.874				0.515; *p* = 0.425				0.393; *p* = 0.410			

^A^, Results obtained based on the total of each category of response Access health service; ^B^, McNemar Browker test; ^C^, X ${}_{calc}^{2}$ -Pearson chi-square association test; *, significant for p < 0.05.

## Covid-19 and the Sample

In the sample, we had two cases of confirmed COVID-19 diagnosis in (P2), with a good outcome. Results regarding relatives and friends who had received the COVID-19 diagnosis were: in (P1), family members (*n* = 0) and friends 25% (*n* = 12); in (P2), family members 25% (*n* = 12) and friends 52% (*n* = 25). Regarding relatives/friends who died due a COVID-19: At (P1) family members (*n* = 0) and friends 14.6% (*n* = 7); in (P2) friends 41.7% (*n* = 20) and family 2% (*n* = 1) (Table 5).

### Open-End Questions

The sample responses to the open-ended questions reported that the worst part of restriction measures and social distancing was related to social isolation (*n* = 26), suspension of PROTIG follow-up (*n* = 13), distance from family/friends (*n* = 21), and financial issues (*n* = 12).

## Discussion

Since the emergence of the new coronavirus (SARS-CoV-2) in China in December 2019, humanity has faced a serious global health crisis. New and numerous cases have quickly emerged in Asian countries such as Thailand, Japan, South Korea, and Singapore, leading the World Health Organization (WHO) to enact a Public Health Emergency of International Importance on January 30, 2020; and an annunciation of a pandemic on March 11, 2020. According to data available on November 28, 2021, 210 countries and territories reported a total of 261 million confirmed cases of COVID-19 and a death estimation of more than 5.2 million people. Although GD care services already faced challenges, the COVID-19 pandemic overloaded the health system, and GD health care became remote, in order to meet demands arising from the limitations for treatment access.

The study on the consequences of COVID-19 in a Brazilian GD population was motivated by previous knowledge about their social vulnerabilities and due to “*open door”* policy for if they needed any help during the period of interrupted assistance. Although the final sample consisted of 48 individuals, study results yield insights into the mental health of the population of transgender women with GD in the pandemic.

### Economical Dimension

During the first period of the pandemic, social isolation was strongly recommended for controlling the virus and to prevent people in risk from developing the full COVID-19 syndrome. However, this important health preventive measure provoked side effects such as the reduction of formal and informal employment worldwide with many workers being unable to work due to the uncertainty concerning COVID-19 (4). In Brazil, this economic crisis did not have catastrophic consequences due to the government's Emergency Aid, established by Law No. 13,982/2020, which provided the transfer of R$ 600.00/month ($ 107 US dollars) for informal and low-income workers (5). This benefit positively affected a large part of the population, especially, the most vulnerable individuals as the patients. It was not without surprise that we faced the truth: many patients got a higher monthly income during the pandemic compared to their previous monthly income.

The living conditions of the patients are extremely stressful; besides the conditions directly associated with the stigma and prejudice due to their GD condition, they live in very precarious economic conditions in general, which expose them to situations of greater marginalization and risk to their physical and mental integrity, corresponds to the description of Minority Stress (6).

### COVID-19 Dimension

The GD population of HCPA has access to specialized care in a highly complex hospital and is viewed by some people as a “privileged group.” During the pandemic period they had to leave their assistance for “a moment in the future,” when it is hoped that a general normalization of attendance at HCPA will occur. Notably, HCPA was and still is a reference center for COVID-19 care in the state and since the last year has prioritized the care of COVID-19 patients.

Even during the difficult scenario with the sudden increase of severe cases with COVID-19, hormonal treatment–used by people with GD to assist in their gender transition–was not seriously impaired (7). The prescriptions of drugs of continuous use (prolonged treatment) were extended by a national resolution in March 2020 which aimed to avoid agglomerations and the interruption of health treatments. This resolution has also allowed for the provision of prescription by e-mail.

The fact that GD patients had to resort to the Emergency Aid is an indication of the worsening social vulnerability during the pandemic. When one observes the need for Emergency Aid associated with mental health situations (depression, irritability, and anxiety) (8) it was identified that the members of the group that needed emergency assistance had more depressive symptoms (9).

Worldwide, the COVID-19 pandemic has led to unprecedented risks for relatively high mental rates of anxiety symptoms (6.33 to 50.9%), depression (14.6 to 48.3%), posttraumatic stress disorder (7 to 53.8%), mental distress (34.43 to 38%), and stress (8.1 to 81.9%) in people worldwide (10). In this study, the patients reported a worsening of mental health due to COVID-19 pandemic stressors. They associated this worse mental health status with social isolation (*n* = 26), suspension of PROTIG follow-up (*n* = 13), distance from family/friends (*n* = 21), and financial issues (*n* = 12). At the same time, the patients suggested alternatives for the continuity of assistance promoted by PROTIG team.

In conclusion, the suspension of systematic health assistance contributed to worsen different dimensions of patients' lives–not only in the health dimension–as we had hypothesized.

We realized that PROTIG team assistance provides more for this population than prescriptions or GAS. The consequences of the pandemic –including the isolation, temporary interruption of occupational activity, and interruption of assistance–provoked a temporary loss of self-esteem (11). This included a loss of belonging to a social group, of hope in the future to achieve the GAS, and the capability to face their own problems–as a mirror image–during group therapy. Since many patients used to complain about the CFM legislation that obligates them to a minimum of 1 year of medical attendance before GAS indication, it was important for the PROTIG team to discover that the work is more important for the patients than we may have wrongly evaluated before.

Another interesting find was the role that families played by providing financial and emotional support during this singular crisis. We characterize families as “groups of people linked by biological, legal or emotional connections” (12). It is still very common that GD patients report prejudice, aggressive attitudes, and misunderstanding by family members regarding gender issues (13, 14). Due to the need for social isolation and continuous care to prevent the proliferation of the virus, we identified reports of an improvement in relationship between the family members and the patients, and also reports of feelings of loneliness when it was not possible to be together.

This improvement in family relations during the pandemic may have been influenced by the change in the paradigm of priorities, when health and survival became more important than sexuality issues.

Fortunately, COVID-19 did not directly reach our sample in a representative way. Initially, only two positive cases were reported, and no deaths, but over time the disease reached friends and family. They also reaffirmed the difficulty/embarrassment and the feeling of internalized or real rejection of access to health services due to their GD condition even during this world health crisis.

## Conclusion

The pandemic scenario affected not only the daily life, but also the social, economic, and health conditions of the world population. With this study, it was possible to verify that the COVID-19 pandemic also affected the population with GD attended at PROTIG.

Regarding our results on the impact of the COVID-19 pandemic on the public population with GD undergoing hospital follow-up, it was possible to identify in the sample, the worsening of social vulnerability factors that underlie strategic actions to access and guarantee health care. The findings reiterate an unstable financial condition with the need to resort to emergency assistance from the Brazilian government.

The research also contributed to the importance of this free public service for this specific population, in addition to demonstrating a significant degree of motivation of these individuals to resume multidisciplinary follow-up through online technologies (telephone, video call).

Considering that the population with GD is already socially vulnerable, individuals in the sample were a negative impacted by the COVID-19 pandemic, but the research also showed two positive points: improved family relationships and the patient's realization of the importance of PROTIG assistance.

## Limitations

This study had some limitations. Firstly, all data was collected with Google Forms. Therefore, the information may vary according to the interpretation/understanding of the patient. Secondly, although our team believed the sample representative, we had almost 1/3 of missing cases, probably due a difficulty in accessing the Internet during the pandemic, a fact that has been reported by several students of the public education institutions in our country. Thirdly, this study did not include a comparative group population. Considering the specificity of GD social vulnerability, the team decided unnecessary to get dates from others groups for comparison purposes. Fourth the study was composed of a sample of transgender men and women who wanted and were able to access care in a specialized service that aims at surgical procedures. Since there is a wide variety of transgenic identities, the sample does not represent all these populations. Lastly, the sample includes transgender men and women who attended at a specific service in Southern Brazil and due to cultural specificities, the results cannot be generalized.

## Data Availability Statement

The original contributions presented in the study are included in the article/Supplementary Material, further inquiries can be directed to the corresponding author.

## Ethics Statement

The studies involving human participants were reviewed and approved by CEP of Hospital de Clínicas de Porto Alegre. The patients/participants provided their written informed consent to participate in this study.

## Author Contributions

FG and ML conceived the idea presented. FG conducted the research protocol. DS helped oversee the project. ML and APVB contributed to the interpretation of the results. KS performed the final review and manuscript submission. All authors discussed the results and contributed to the final manuscript.

## Funding

This study was supported by the *Fundo de Incentivo a Pesquisa e Eventos do Hospital de Cl*í*nicas de Porto Alegre* (FIPE/HCPA), Fundação de Amparo à Pesquisa do Estado do Rio Grande do Sul (grant number INCT/FAPERGS: 17/2551-0000519-8), National Council for Scientific and Technological Development (CNPq), Coordination for the Improvement of Higher Education (CAPES) and Pos Graduate Program in Behavioral Sciences, Psychiatry at UFRGS. This study was financed in part by the Coordination for the Improvement of Higher Education Personnel—Brazil (CAPES)—Finance Code 001.

## Conflict of Interest

The authors declare that the research was conducted in the absence of any commercial or financial relationships that could be construed as a potential conflict of interest.

## Publisher's Note

All claims expressed in this article are solely those of the authors and do not necessarily represent those of their affiliated organizations, or those of the publisher, the editors and the reviewers. Any product that may be evaluated in this article, or claim that may be made by its manufacturer, is not guaranteed or endorsed by the publisher.
