# Corrigendum: The impact of the COVID-19 pandemic on the lives of people with Gender Dysphoria

**DOI:** 10.3389/fpubh.2023.1239717

**Published:** 2023-07-10

**Authors:** Fernanda Guadagnin, Dhiordan Cardoso da Silva, Karine Schwarz, Anna Paula Villas Bôas, Maria Inês Rodrigues Lobato

**Affiliations:** ^1^Clinical Hospital of Porto Alegre, Porto Alegre, Brazil; ^2^Department of Psychiatry, Gender Identity Program at Hospital de Clínicas de Porto Alegre, Porto Alegre, Rio Grande do Sul, Brazil; ^3^Postgraduate Program in Medical Sciences: Endocrinology, Federal University of Rio Grande do Sul (UFRGS), Porto Alegre, Brazil; ^4^Postgraduate Program in Psychiatry and Behavioral Sciences at the Federal University of Rio Grande do Sul, Porto Alegre, Brazil

**Keywords:** Gender Dysphoria, COVID-19, health monitoring, vulnerability, transgender

In the published article, there was an error in author list and affiliations. They originally stated: “Fernanda Guadagnin^1^, Dhiordan Cardoso da Silva^2^, Karine Schwarz^2^^*^, Anna Paula Villas Bôas^2^ and Maria Inês Rodrigues Lobato^1^

^1^ Clinical Hospital of Porto Alegre, Porto Alegre, Brazil

^2^ Department of Psychiatry, Gender Identity Program at Hospital de Clínicas de Porto Alegre, Rio Grande do Sul, Porto Alegre, Brazil”

The correct author list and affiliations appear below: “Fernanda Guadagnin^1^, Dhiordan Cardoso da Silva^2^, Karine Schwarz^2, 3*^, Anna Paula Villas Bôas^2^ and Maria Inês Rodrigues Lobato^1, 4^

^1^ Clinical Hospital of Porto Alegre, Porto Alegre, Brazil

^2^ Department of Psychiatry, Gender Identity Program at Hospital de Clínicas de Porto Alegre, Porto Alegre, Rio Grande do Sul, Brazil

^3^ Postgraduate Program in Medical Sciences: Endocrinology, Federal University of Rio Grande do Sul (UFRGS), Porto Alegre, Brazil

^4^ Postgraduate Program in Psychiatry and Behavioral Sciences at the Federal University of Rio Grande do Sul, Porto Alegre, Brazil”

In the published article, there was an error in the **Funding** statement. The funding statement for the Coordination for the Improvement of Higher Education (CAPES) was without the funding code. It originally stated: “This study was supported by the *Fundo de Incentivo a Pesquisa e Eventos do Hospital de Cl*í*nicas de Porto Alegre* (FIPE/HCPA), Fundação de Amparo à Pesquisa do Estado do Rio Grande do Sul (Grant Number INCT/FAPERGS: 17/2551-0000519-8), National Council for Scientific and Technological Development (CNPq), Coordination for the Improvement of Higher Education (CAPES) and Pos Graduate Program in Behavioral Sciences, Psychiatry at UFRGS.”

The correct **Funding** statement appears below:

