# Supplementary material for: The Impact of the COVID-19 Pandemic on the Lives of People With Gender Dysphoria
Source: Front Public Health. 2022 Jul 6;10:878348. doi: 10.3389/fpubh.2022.878348 (PMC9298877; doi:10.3389/fpubh.2022.878348)
Supplement: Supplementary file 1 [file Table_1.DOCX]

**INVENTORY**

Do you agree to participate in the research?

( ) Yes ( ) No

Features

Hello. Let’s start the questions with information about you.

In which state do you live:

1. Rio Grande do Sul RS
2. Ceará CE
3. Federal District DF
4. Minas Gerais MG
5. Paraná PR
6. Santa Catarina SC
7. Sao Paulo SP
8. Other

You consider yourself:

( ) White

( ) Black

( ) Mixed

( ) Indigenous

( ) Yellow

What’s your education?

( ) Unliterate (a)

( ) Incomplete Elementary School

( ) Complete Elementary School

( ) Incomplete High School

( ) Complete High School

( ) Higher Education

( ) Post-Graduation

Work and income

Now the questions will be about your work and your income.

Your working condition BEFORE COVID-19:

( ) Employee with work permit

( ) Employee without a work permit/ self-employed

( ) Unemployed

Your CURRENT work situation:

( ) Employee with work permit

( ) Employee without a work permit/ self-employed

( ) Unemployed

BEFORE the start of the COVID-19 pandemic, your monthly income was:

( ) No income

( ) Up to R$ 1,045.00 (one minimum wage)

( ) from R$ 1,045.01 to R$ 2,090.00 (between 1 and 2 minimum wages)

( ) from R$ 2,090.01 to R$ 3,135.00 (between 2 and 3 minimum wages)

( ) from R$ 3,135.01 to R$ 5,225.00 (between 3 and 5 minimum wages)

( ) from R$ 5,225.01 to R$ 10,450.00 (between 5 and 10 minimum wages)

( ) Other

AFTER the start of the COVID-19 pandemic, what was your monthly income?

( ) No income

( ) Up to R$ 1,045.00 (one minimum wage)

( ) from R$ 1,045.01 to R$ 2,090.00 (between 1 and 2 minimum wages)

( ) from R$ 2,090.01 to R$ 3,135.00 (between 2 and 3 minimum wages)

( ) from R$ 3,135.01 to R$ 5,225.00 (between 3 and 5 minimum wages)

( ) from R$ 5,225.01 to R$ 10,450.00 (between 5 and 10 minimum wages)

( ) Other

Did you have to apply for the government’s emergency aid due to COVID-19?

( ) Yes ( ) No

Have you been approved for the benefit you applied for?

( ) Yes ( ) No

Gender identity and sexual orientation

Now the questions will be about gender, sexual orientation, and treatment at PROTIG.

What’s your designated gender at birth?

( ) Man

( ) Women

What’s your sexual orientation?

( ) Attracted to women

( ) Attracted to men

( ) Attracted to men and women

What type of hormone treatment do you currently take?

( ) Hormonal masculinizing

( ) Hormonal feminizing

( ) Hormonal block

( ) I have never had hormone treatment

( ) I abandoned hormone treatment

COVID-19

The last block, below are more specific questions about the COVID-19 pandemic and health services.

Have you been diagnosed with COVID-19 confirmed?

( ) Yes ( ) No

Have you sought any health services due to COVID-19 symptoms?

( ) Yes ( ) No

If you have sought health services, has being “trans” hindered your access to care?

( ) Yes ( ) No

How was your relationship with your family BEFORE the pandemic?

( ) Good

( ) Bad

How is the relationship with your family after the onset of the pandemic?

( ) Good

( ) Bad

Do you have people close to you diagnosed with COVID-19?

( ) No

( ) Family (father/mother/siblings/children)

( ) Friends

Do you have people close to you who died because of COVID-19?

( ) No

( ) Family (father/mother/siblings/children)

( ) Friends

At this point in the pandemic you feel:

( ) Anxious

( ) Depressed

( ) Angry

What is the worst part of social isolation, for you:

What do you intend to do after isolation (first wish)?

Would you like to add something that hasn’t been asked?
